# Genomic Analysis of Immune Response against *Vibrio cholerae* Hemolysin in *Caenorhabditis elegans*

**DOI:** 10.1371/journal.pone.0038200

**Published:** 2012-05-31

**Authors:** Surasri N. Sahu, Jada Lewis, Isha Patel, Serdar Bozdag, Jeong H. Lee, Joseph E. LeClerc, Hediye Nese Cinar

**Affiliations:** 1 Division of Virulence Assessment, Food and Drug Administration, Laurel, Maryland, United States of America; 2 Division of Molecular Biology, Food and Drug Administration, Laurel, Maryland, United States of America; 3 Neuro-Oncology Branch, National Cancer Institute, National Institutes of Health, Bethesda, Maryland, United States of America; 4 Oak Ridge Institute for Science and Education, Oak Ridge, Tennessee, United States of America; 5 Kyungpook National University (KNU), Daegu, South Korea; University of Minho, Portugal

## Abstract

*Vibrio cholerae* cytolysin (VCC) is among the accessory *V. cholerae* virulence factors that may contribute to disease pathogenesis in humans. VCC, encoded by *hlyA* gene, belongs to the most common class of bacterial toxins, known as pore-forming toxins (PFTs). *V. cholerae* infects and kills *Caenorhabditis elegans* via cholerae toxin independent manner. VCC is required for the lethality, growth retardation and intestinal cell vacuolation during the infection. However, little is known about the host gene expression responses against VCC. To address this question we performed a microarray study in *C. elegans* exposed to *V. cholerae* strains with intact and deleted *hlyA* genes.

Many of the VCC regulated genes identified, including C-type lectins, Prion-like (glutamine [Q]/asparagine [N]-rich)-domain containing genes, genes regulated by insulin/IGF-1-mediated signaling (IIS) pathway, were previously reported as mediators of innate immune response against other bacteria in *C. elegans*. Protective function of the subset of the genes up-regulated by VCC was confirmed using RNAi. By means of a machine learning algorithm called FastMEDUSA, we identified several putative VCC induced immune regulatory transcriptional factors and transcription factor binding motifs. Our results suggest that VCC is a major virulence factor, which induces a wide variety of immune response- related genes during *V. cholerae* infection in *C. elegans*.

## Introduction

*V. cholerae* cytolysin (VCC) is among the accessory *V. cholerae* virulence factors that may contribute to the sporadic form of diarrheal disease pathogenesis. VCC, encoded by *hlyA* gene, belongs to the most common class of bacterial toxins, the pore-forming toxins (PFTs), which are important virulence factors. Most of the O1 biotype El Tor, O139, and non-O1/non-O139 *V. cholerae* isolates, produce a 80-kD water soluble cytolysin (VCC) [1]–[3]. VCC causes tissue and cell damage through apoptosis, autophagy, cellular vacuolization, cell lysis and necrosis. [4]–[9]. Studies using host models such as infant mouse, rabbit ileal loop, streptomycin fed adult C57BL/6 mice models, and nematode *C. elegans* infection model, suggest that VCC was responsible for the residual toxicity observed with some of the vaccine strains with full or partial coding sequences of *hlyA* gene [4], [10], [11].

*Caenorhabditis elegans* has been used as an invertebrate host model to identify and assess virulence factors of several human pathogens including *V. cholerae* [11]–[14]. *V. cholerae* causes lethal infection in the nematode *Caenorhabditis elegans* via a cholera toxin (Ctx) and toxin co-regulated pili (Tcp) independent process, providing a useful host model system to screen for the virulence factors other than Ctx and Tcp. Worm lethality effect inflicted by *V. cholerae*, is mediated by LuxO-regulated genes in the quorum sensing (QS) pathway, such as *hapR*, *V. cholerae* metalloprotease gene *PrtV* [14], and VCC encoding gene *hlyA* [11]. *hlyA* also causes developmental delay and intestinal vacuolation in *C. elegans* [11].

Host responses to VCC at the molecular level, and the significance of these responses in host organisms' defense during *V. cholerae* pathogenesis, remain poorly understood. *C. elegans* provides an excellent model to address these questions. Here we report our findings regarding genome wide host transcriptional response to VCC in *C. elegans* during *V. cholerae* infection. We performed a microarray study in *C. elegans* which was exposed to *V. cholerae* strains with intact and deleted *hlyA* genes for 18 hours. Expression profiles of the worms exposed to *hlyA*(−) *V. cholerae* strains were compared with the expression profiles of the worms exposed to *hlyA*(+) *V. cholerae* strains. Many of the differentially expressed genes previously reported as mediators of innate immune response against other bacteria in *C. elegans*, suggesting that *C. elegans* uses common and specific mechanisms against *V.cholerae* and these defenses are induced by VCC. Among the differentially expressed genes are: C-type lectins, *abu* (activated in blocked unfolded protein response) genes, which contain Prion-like (glutamine [Q]/asparagine[N]-rich)-domain, and genes regulated by *daf-16*. Immune response function of the subset of the differentially expressed genes against *V. cholerae* infection was confirmed using RNAi. Using a machine learning algorithm called FastMEDUSA, we identified putative immune regulatory transcriptional factors, which are regulated by VCC. FastMEDUSA was also used to discover the transcription factor binding motifs, which were later analyzed using the GOMO (Gene Ontology for Motifs) tool to identify the GO-terms associated with these motifs. Go terms related to pathogen recognition and to immune and inflammatory responses, were found to be significantly associated with the motifs identified using FastMEDUSA.

## Materials and Methods

### Bacterial strains, media and culture conditions

Bacterial strains used in this study include: E7946: *V. cholerae* Wild-type O1 El Tor, Ogawa strain, HNC45: E7946 Δ*hlyA*, CVD 109: Δ(*ctxAB zot ace*) of parental strain E7946, CVD110 Δ*(ctxAB zot ace) hlyA::*(*ctxB mer*) *Hgr* of parental strain E7946.

*V. cholerae* strains were cultured in tryptic soy broth (TSB, Becton Dicson Microbiology System, BBL, Cockeysville, MD) media supplemented with 1% NaCl at 30°C. *E.coli* OP 50 was grown in LB culture media.

### *C. elegans* strains, maintenance and microscopy

Strains N2, NL2099 *rrf-3 (pk1426)*, GR1373 *eri-1 (mg366)*, RB711 *pqm-1(ok485)*, VC2169 *abu-15(ok2878)*, VC1806 *nhr-234(gk865)*, RB1590 *pax-1 (ok1949)*, VC1204 *nhr-34 (gk556)*, RB657 *nhr-23 (ok407)*, RB1044 *Y47H9C.2 (ok990)*, and SAL139 *pha-1(e2123); denEx17 [dod-22::GFP+pha-1(+)]* were acquired from the *Caenorhabditis* Genetics Center (CGC). All the strains were maintained at 22°C except GR1373 (*eri-1*), which is maintained at 16°C. The wild type Bristol strain N2, was cultured in *C. elegans* habitation media (CeHM) in tissue culture flasks on a platform shaker [15]. Nematodes were bleached (0.5 M NaOH, 1% Hypochlorite) to collect eggs which were incubated in M9 media for 24 hours to bring them to synchronized L1 stage and then transferred to CeHM. For microscopy, mixed stage worms grown on OP50 and test bacteria seeded nematode growth media (NGM) plates, were imaged on a Leica MZ16FA stereomicroscope.

### RNA Isolation

Synchronized L1 stage N2 animals were transferred to *C. elegans* habitation media (CeHM) and incubated for 24 hours. Animals were washed with M9 buffer and transferred to NGM plates containing *E. coli* strain OP50, *V. cholerae* strains E7946, E7946 *Δ hly*, CVD109, and CVD110, and incubated at 22°C for 18 hours. Worms were collected and washed in M9 buffer and RNA was extracted using TRIzol reagent (Invitrogen). Residual genomic DNA was removed by DNase treatment (Ambion, Austin, TX). Three independent RNA isolations were performed with each condition for microarray analysis.

### Microarray Analysis

For each experimental condition, RNA was isolated from three biological replicate samples. cRNA was synthesized from 10 µg of total RNA, and samples were hybridized to the *C. elegans* GeneChip (Affymetrix, Santa Clara, CA) by the FDA/CFSAN/DMB Microarray Facility following the manufacturers instruction. The chip represents 22500 transcripts of the expressed *C. elegans* genome based on the December 2005 genome sequence. The data were processed using Partek Genomics Suite, version 6.4 Partek Inc, St. Louis, MO. The robust multichip averaging algorithm was used to normalize and summarize the probe data into probe set expression values. Analysis of variance, fold-change, and false discovery rate (FDR) calculations were also performed using Partek® Genomics Suite TM version 6.5 (Copyright © 2010 Partek Inc., St. Louis, MO, USA). Transcripts showing a corrected *p* value of <0.05 and fold change ≤−1.2 or ≥1.2 were considered differentially expressed between experimental treatments groups. The microarray data have been deposited in the Microarray Informatics, EMBL. Accession number is E-TABM-840.

### Functional Enrichment Analysis

Genes showing a significant change in expression by microarray analysis (*p*<0.05) were analyzed using R software (R Development Core Team (2009): A language and environment for statistical computing. R Foundation for Statistical Computing, Vienna, Austria. ISBN 3-900051-07-0, URL http://www.R-project.org). Genes were compared against a 22,500 *C. elegans* gene data base to identify over-represented Gene Ontology terms (Table S1). Statistical analysis was performed using the chi-square test and the Yates' continuity correction. Significant functional terms were defined as *p*<0.05.

### qRT-PCR

cDNA was synthesized from 5 µg of total RNA using random hexamers and SuperScript II reverse transcriptase (Invitrogen). qRT-PCR was performed using SYBR Advantage quantitative PCR premix (Clontech) and gene-specific oligonucleotide primers on the LightCycler (BIO RAD). Primers for qRT-PCR are following: *clec-7*: (fwd) ttggctgttgtaggcaatca, (rev) tcactgggaatccgttatcc; *fmo-2*: (fwd) tgctgtcataggagctggtg, (rev) catctgacgcctcaaaacaa; *clec-46*: (fwd) cttcctcggttcttgcactt, (rev) gcggtttccaacaaaaacac; *C23G10.1*: (fwd) ccatccactcttggttgctt, (rev) tcacgtgctcctttttcctt; *col-41*: (fwd) caccaggaactccaggaaac, (rev) gtggggttctgtcgtcttgt; *B0024.4*: (fwd) caacaacattgagcgcagag, (rev) tgtgtagtcgtctgttggaacc; *ttr-21*: (fwd) tgtgtcaaggacaaccagcta, (rev) ttccagcaactcgaaaggtt; *dct-5*: (fwd) gctgcaaaatgtggaaatga, (rev) aagttttgggcacagtccag; *pqn-5*: (fwd) gctcagccacaacaaactca, (rev) ctggcactgttgctgacatt.

Relative fold-changes for transcripts were calculated using the comparative *C_T_* (2^−ΔΔ*CT*^) method [16]. Cycle thresholds of amplification were determined by Light Cycler software (BIO RAD). All samples were run in triplicates and normalized to internal control.

### RNA Interference

*E. coli* DH5α bacterial strains expressing double-stranded *C. elegans* RNA [17] were grown in LB broth containing ampicillin (100 µg/ml) at 37°C and plated onto NGM containing 100 µg/ml ampicillin and 1 mM isopropyl 1-thio-β-d-galactopyranoside (IPTG). RNAi-expressing bacteria were allowed to grow overnight at 37°C. Synchronized L1 stage NL2099 (*rrf-3*) or GR1373 (*eri-1*) strains were used for RNAi experiments. NL2099 (*rrf-3*) has been used for the functional validation of the differentially expressed genes identified through microarray, and GR1373 (*eri-1*) for the rest of the RNAi experiments regarding prion-like (Q/N rich) domain protein genes, and FastMedusa identified genes. NL2099 (*rrf-3*) worms were exposed to fresh RNAi expressing bacterial lawn on NGM media for 48 hours, then washed with M9 and plated on NGM plates containing *V. cholerae* wild type E7946, E7946 *Δhly*, or *E.coli* OP50 bacterial lawn, and incubated at 22°C. GR1373 (*eri-1*) worms were initially treated with *V. cholerae* wild type E7946, E7946 *Δhly* or *E.coli* OP50 bacterial lawn and incubated first at 16°C for 24 hours followed by incubation at 25°C for next 24 hours. Worms were than transferred to the RNAi bacterial lawn and incubated at 25°C for the rest of the experiment. L4440 RNAi which contains the empty vector was included as a control in all experiments.

### *C. elegans* Survival Analysis

Pathogen lawns for survival assays along with food bacteria OP50 were prepared by inoculating NGM (in 6-cm Petri plates) with 50 µl of an overnight bacterial culture. Plates were incubated overnight at room temperature before animals were added. Worms treated with RNAi bacteria, or mutant worms to be tested were transferred to NGM plates containing *V. cholerae* wild type E7946, E7946 *Δhly* or *E. coli* OP 50 bacterial lawns and incubated at 22°C with ∼20–30 L4 stage worms added to each plate. Animals were scored every 24 h for survival and transferred to fresh bacterial lawns everyday to avoid confusion with progeny. Animal survival was plotted using Kaplan-Meier survival curves and analyzed by log rank test using GraphPad Prism (GraphPad Software, Inc., La Jolla, CA). Survival curves resulting in *p* values of <0.05 relative to control were considered significantly different.

### FastMEDUSA analysis

We used FastMEDUSA software to discover experimental condition-specific transcription factors (TFs) and motifs in *C. elegans* [18]. FastMEDUSA is a parallelized version of MEDUSA algorithm [19], which trains a model from expression and promoter sequences of genes in a number of experimental conditions. We analyzed the FastMEDUSA model to extract condition-specific significant TFs and motifs in *C. elegans*.

FastMEDUSA requires discretized gene expression profiles. To this end, we discretized gene expression data by using E7946 samples as reference. We computed differentially expressed genes (DEGs) by using ANOVA (FDR≤0.05) in Partek® Genomics SuiteTM version 6.5 (Copyright © 2010 Partek Inc., St. Louis, MO, USA). For each DEG in a sample, we computed the ratio to its median expression signal across reference samples. A gene in a sample was called *upregulated* if the ratio ≥1.0 and *downregulated* otherwise. Genes that had inconsistent expression calls across technical replicates were filtered out. We obtained a list of candidate TFs in *C. elegans* from EDGEdb [20], and 1,000 bp promoter sequence of all DEGs from BioMart [21].

We ran FastMEDUSA five times on Biowulf cluster at the National Institutes of Health. For each run, we computed significance score of TFs as described in [19] and selected the top 30 significant TFs for each condition. We selected consensus significant TFs that occur in the top list in all runs. We computed significant motifs similarly.

FastMEDUSA applies a machine learning algorithm called boosting [22] to train a predictive model as an alternating decision tree. In order to determine how many boosting iterations are needed to train a model, we ran FastMEDUSA on 90% of the input data and tested the model on the remaining 10%. Running FastMEDUSA with 800 boosting iterations was optimal to learn the model for this data set. When building the model, if the boosting algorithm gives the same score for more than one transcription factor or motif, FastMEDUSA makes a random choice. In other words, FastMEDUSA potentially builds a different model at each run. Thus, we ran FastMEDUSA five times using a different random seed value at each run and selected TFs and motifs that are overrepresented in these models.

### Semi-quantitative RT-PCR analysis for V. cholerae virulence genes

The relative transcript abundance and expression of *V.cholerae* virulence genes at different temperatures was evaluated using semi-quantitative RT-PCR in *V. cholerae* wild type strain E7946. Cultures of E7946 were grown at 16°C, 22°C, 30°C, and 37°C by shaking at 120 rpm, in TSB with 1% NaCl. Primers for semi-quantitative RT-PCR are following: *hlyA*: (fwd) TGAGCGTAATGCGAAGAATG, (rev) GCGGGCTAATACGGTTTACA; *ace*: (fwd) GATGGCTTTACGTGGCTTGT, (rev) AAGCCGCTGTATTGAGGAGA; *ctxA*: (fwd) TATAGCCACTGCACCCAACA, (rev) CAAGCACCCCAAAATGAACT; *zot*: (fwd) AGCTTTGAGGTGGCTTTTGA, (rev) GGTAAACTTTGCCCCTAGCC; control gene *sanA*: (fwd) TTGCTGTGGCTGACTATTGG, (rev) CCAATACCACTGCAACCTGA. First strand cDNA was synthesized by using random hexamers (Invitrogen SuperScriptTM III First-Strand Synthesis System for RT-PCR) according to manufacturer protocol. 1 µl aliquot of resulting cDNA was used in each PCR reaction. Fragments were amplified by 30 cycles of PCR (94°C for 30 sec, 52°C for 30 sec and 72°C for 30 sec. After amplification, 1 µl PCR product was run on a 1.5% agarose gel. The gel image was photographed by video gel documentation system (Universal Hood II-S.N. 76S/04668 by BIO-RAD Laboratories). The semi-quantitative RT-PCR images were evaluated and pixels in respective bands were quantified using Adobe Photoshop and ImageJ (by NIH).

## Results and Discussion

### Transcriptional response to *V. cholerae* cytolysin (VCC) during infection in *C. elegans*

**Figure 1:**
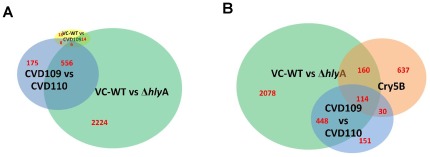
Genome-wide expression profile comparisons of the *C. elegans* genes regulated by pore forming toxins: *V. cholerae hlyA*, and *B. thurigiensis* Cry5B. (A) A Venn diagram illustrating number of genes expressed in CVD109 versus CVD110, *V. cholerae* wild type versus *DhlyA*, and *V. cholerae* wild type versus CVD109 comparisons. (B) A Venn diagram illustrating number of genes expressed in CVD109 versus CVD110, *V. cholerae* wild type versus DhlyA, and Cry5B induction comparisons.

Host responses to VCC at the molecular level, and the significance of these responses in host defense during *V. cholerae* pathogenesis, remain poorly understood. To address this question, we performed a microarray study of *C. elegans*, which was exposed to *V. cholerae* strains with intact and deleted *hlyA* genes using Affymetrix *C. elegans* arrays. Gene expression in worms exposed to Wild type O1 el tor *V. cholerae* strain E7946 was compared with gene expression in worms exposed to the *hlyA* deletion mutant of E7946. We also compared gene expressions between vaccine strains CVD110 and CVD109 [23], both of which were generated in the E7946 genetic background. In the vaccine strain CVD109 the virulence genes *zot, ace, ctxA, ctxB* are deleted, but *hlyA* locus is intact. CVD110 was generated in the CVD109 genetic background by inactivating the *hlyA* gene via *ctxB* insertion [23]. We identified 2,800 differentially expressed genes when we compared expression in *C. elegans* exposed to *V. cholerae* wild type strain E7946, versus E7946 *Δhly*, and 743 differentially expressed genes when we compared expression in *C. elegans* exposed to nearly isogenic *V. cholerae* vaccine strains CVD109 (*hlyA+*), versus CVD110 (*hlyA−*) [considering fold change (+/−) 1.2, FDR = 0.5 and P<0.01]. Microarray data were confirmed using qRT-PCR to measure the expression levels of a set of selected genes (Fig. S1). We found significant overlap between “CVD109 versus CVD110” and “E7946 versus *ΔhlyA*” comparisons such that there were 562 genes in common between the two (Fig. 1A). A possible explanation for the dissimilarity in the differentially expressed gene number is that presence of the inserted *ctxB* gene in the CVD110 strain. *ctxB*, a known immunomodulator, was inserted as an immunologic adjuvant to enhance immune responses against the CVD110 vaccine strain [23]. Hence, over expression of *ctxB* in CVD110 may cancel out some of the immune response genes induced by hemolysin in CVD109, leading determination of lower number of genes in CVD109 versus CVD110 data set.

Induction by wild type *V. cholerae* strain E7946 versus *V. cholerae* vaccine strain with a deleted virulence cassette but intact *hlyA*, CVD109, revealed only 44 differentially expressed genes [considering fold change (+/−) 1.2, FDR = 0.5 and P<0.01]. This suggests that *ctxA*, *ctxB*, *ace* and *zot* genes of *V. cholerae* are not involved in immune response induction, and VCC is the major virulence factor inducing immune response during *V. cholerae* infection in *C. elegans* (Fig. 1A). Our findings support previous studies that showed cholerae toxin (CTX) may not be involved in the *V. cholerae* pathogenesis in *C. elegans* [11], [14,14].

### Genome wide transcriptional responses to *V. cholerae* hemolysin and *B. thuringiensis* crystal toxin show significant overlap

VCC shows structural similarity to *Bacillus thuringiensis* crystal toxin, which is also a “small-pore” pore forming toxin [24], [25]. Previous studies revealed that *B. thuringiensis* crystal toxin causes intestinal damage and lethality in *C. elegans* [26]. We compared genome wide transcriptional responses to *B. thuringiensis* crystal toxin [27], with our data regarding transcriptional response to VCC and found a significant overlap between the two genomic responses (Fig. 1B). These results suggest that pore forming toxins induce immune responses in the host organisms through common and specific mechanisms. Since pore forming toxins are the most common bacterial toxins, we speculate that they might contribute to the induction of an immune response during a wide range of bacterial infections.

### Previously reported *C. elegans* innate immune response genes are among the hemolysin responsive genes detected in our microarray analysis

Table 1 shows the list of genes up-regulated ≥2 fold in both ‘E7946 over E7946Δ*hlyA*’ and ‘CVD109 over CVD110’. About one third of the differentially expressed top ranker genes previously reported to be mediators of innate immune response against other bacteria in *C. elegans* (Table 1). C-type lectins, such as *clec-45, clec-174, clec-209, clec-17, clec-47*, are among the differentially expressed top ranker genes. C-type lectins have been proposed to act as pathogen-recognition molecules, and/or as effectors of the antimicrobial response by binding to carbohydrates on the surface of pathogens. The *abu* (activated in blocked unfolded protein response) genes, which contain a Prion-like (Q/N-rich)-domain, such as *pqn-5, abu-6, abu-7, abu-8*, were enriched in the Table 1. Lipase related gene *lips-6*, tollish gene *toh-1*, genes regulated by *daf-16*; *dod-22* and *dod-24*, were also among the high rankers listed in this table.

**Table 1:** Genes induced over twofold following infection of *C. elegans* with *hly(+) V. cholerae* strains.

Gene Name	Description	CVD109/CVD110 fold change	DhlyA/E7946 fold change	Reported Immune response function
*clec-45*	C-type Lectin	14.4	17.8	Schulenburg et al, 2007
*clec-174*	C-type Lectin	9.9	10.1	Schulenburg et al, 2007
*C23G10.11*	hypothetical protein/Confirmed	6.0	9.7	Shapira et al, 2006
*fmo-2*	Flavin-containing MonoOxygenase family	2.3	8.9	Irazoqui et al, 2008
*dct-5*	DAF-16/FOXO Controlled, germline Tumor affecting	4.7	7.9	
*col-41*	Collagen	5.7	6.2	
*col-90*	Collagen	6.5	5.8	
*grd-6*	Groundhog (hedgehog-like family)	3.6	5.6	
*B0024.4*	hypothetical protein/Confirmed	3.0	5.2	
*F35B3.4*	hypothetical protein/Confirmed	3.9	4.9	
*F55G11.4*	hypothetical protein/Confirmed	3.7	4.5	Alper et al, 2007
*Y47D7A.13*	hypothetical protein/Partially confirmed	3.2	4.2	
*col-54*	Collagen	3.6	4.1	
*C25H3.10*	hypothetical protein	2.6	4.1	
*pqn-5*	Prion-like-(Q/N-rich)-domain-bearing protein	3.3	3.9	Russell et al, 2008
*C42D4.3*	hypothetical protein/Confirmed	2.5	3.7	
*abu-6*	Activated in Blocked Unfolded protein response	3.2	3.6	Russell et al, 2008
*C50F7.5*	hypothetical protein/Partially confirmed	2.8	3.6	
*F53A9.2*	hypothetical protein/Confirmed	2.3	3.5	
*F49H6.13*	hypothetical protein/Partially confirmed	3.2	3.4	
*abu-8*	Activated in Blocked Unfolded protein response	2.5	3.2	Russell et al, 2008
*toh-1*	Tollish (Tolloid and BMP-1 family)	2.1	3.1	
*abu-7*	Activated in Blocked Unfolded protein response	2.9	3.1	Russell et al, 2008
*lips-6*	Lipase related	5.1	3.1	
*F22H10.2*	hypothetical protein/Partially confirmed	2.2	3.0	
*C35C5.8*	hypothetical protein	2.5	3.0	
*F44G3.10*	hypothetical protein/Confirmed	2.2	2.9	
*col-156*	Collagen	3.0	2.8	
*dod-24*	Downstream Of DAF-16 (regulated by DAF-16)	3.0	2.8	Troemel et al, 2006Styer et al, 2008
*toh-1*	Tollish (Tolloid and BMP-1 family)	2.0	2.8	
*clec-209*	C-type Lectin	3.7	2.8	Schulenburg et al, 2007
*T22F3.11*	hypothetical protein	2.1	2.8	
*glc-1*	Glutamate-gated ChLoride channel	2.1	2.7	
*ZK180.5*	hypothetical protein	4.0	2.7	
*Y54G2A.11*	hypothetical protein	2.4	2.7	
*dod-22*	Downstream Of DAF-16 (regulated by DAF-16)	2.1	2.5	Shapira et al, 2006Alper et al, 2007
*clec-17*	C-type Lectin	2.8	2.5	O'Rourke et al, 2006Schulenburg et al, 2007
*Y95B8A.2*	hypothetical protein/Confirmed	2.4	2.5	
*grd-14*	Groundhog (hedgehog-like family)	3.6	2.4	
*nspb-12*	Nematode Specific Peptide family, group B	2.5	2.4	
*F54B8.4*	hypothetical protein/Partially_confirmed	2.1	2.4	
*clec-47*	C-type Lectin	2.3	2.4	Schulenburg et al, 2007
*lgc-21*	Ligand-Gated ion Channel	3.2	2.4	
*F53A9.8*	hypothetical protein/Confirmed	2.5	2.3	O'Rourke et al, 2006
*C05E7.2*	hypothetical protein/Partially_confirmed	2.6	2.3	
*D2096.6*	hypothetical protein/Partially_confirmed	2.7	2.3	
*Y42A5A.3*	hypothetical protein/Confirmed	2.0	2.3	Troemel et al, 2006
*C06E8.5*	bacterial permeability-increasing protein	2.0	2.2	
*C28H8.5*	hypothetical protein	4.3	2.2	
*F41E6.11*	hypothetical protein/Partially_confirmed	2.2	2.1	
*nspb-11*	Nematode Specific Peptide family, group B	2.3	2.1	
*T12D8.5*	hypothetical protein/Confirmed	2.0	2.1	
*ZK1307.2*	hypothetical protein/Confirmed	2.3	2.1	
*T22B2.6*	hypothetical protein/Confirmed	2.5	2.1	
*mup-4*	Muscle Positioning	2.7	2.0	

Only, genes induced over twofold in both CVD109/CVD110 and *ΔhlyA*/E7946 comparisons are listed.

### The immune response function of the subset of the genes up regulated against *V. cholerae* hemolysin was confirmed using RNAi

**Figure 2:**
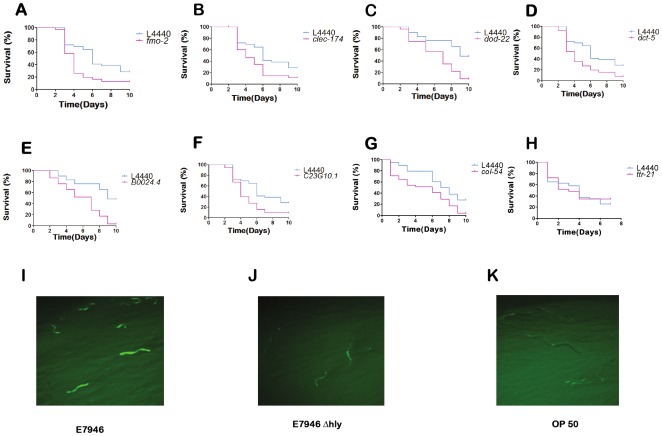
VCC- induced *C. elegans* genes mediate immune response. (A) *fmo-2*, *p* = 0.0063 (B) *clec-174*, *p* = 0.0135 (C) *dod-22*, *p* = 0.0007 (D) *dct-5*, *p* = 0.0045 (E) *B0024.4*, *p* = 0.0001(F) *C23G10.1*, *p* = 0.0038 (G) *col-54*, *p* = 0.0001 (H) *ttr-21*, *p* = 0.7436, RNAi result in lethality. Expression of *dod-22::GFP* in worms fed on (I) *V. cholerae* wild type strain E7946, (J) *DhlyA*, and (K) OP50.

Seven, out of the nine genes tested, caused increased lethality when knocked down using RNAi (Fig. 2). These data suggest that these genes may have immune response functions. Some of these genes such as *fmo-2*, *clec-174*, and *dod-22* were previously reported as immune response genes in *C. elegans* against other bacterial species. The flavin-containing MonoOxigenase family gene *fmo-2* expression is up-regulated against *Staphylococcus aureus* through the β-catenin pathway [28]. The C-type lectin family gene *clec-174* was shown to be up-regulated during *Pseudomonas aureginosa* and *Photorhabdus luminescens* infections in *C. elegans* [29]. *dod-22*, a CUB domain containing gene, was previously shown to be involved in the immune response against gram negative organisms *S. Marcescens* and *P. aeruginosa* via *nsy-1* MAP kinase and *daf-16* insulin signaling pathways [30]. Expression of the *dod-22* gene is regulated by the insulin signaling pathway gene *daf-16* [31]. We found that *dod-22::GFP* expression is induced in the *C. elegans* intestine during *V. cholerae* infection, and this induction is *hlyA* dependent (Fig. 2I, 2J, 2K). *dct-5* was identified as a direct DAF-16 target [32], and found to regulate tumor growth in *C. elegans* [33]. B0024.4 and C23G10.1 genes were not previously characterized. B0024.4 gene encodes a putative glycoprotein, and C23G10.1 gene encodes a serine/threonine specific protein phosphatase PP1, with 64.9% similarity to human serine/threonine-protein phosphatase PP1-alpha catalytic subunit. *col-54* encodes a protein similar to type IV and type XIII collagens which are located in basement membranes. We found that *col-54* RNAi causes increased lethality in *C. elegans* exposed to *V. cholerae* wild type strain E7946 (Fig. 2G). Recent studies reported a dose-dependent antimicrobial activity of extracellular matrix collagens against group A, C, and G streptococci [34], [35]. Our data suggest a previously unrecognized innate immune response function for *col-54*, against *V. cholerae*.

Altogether our microarray and RNAi experiments indicate that *V. cholerae* hemolysin induces a variety of immune response genes in *C. elegans* during *V. cholerae* infection.

### PQN/ABU Unfolded Protein response (UPR) genes are regulated through VCC

**Figure 3:**
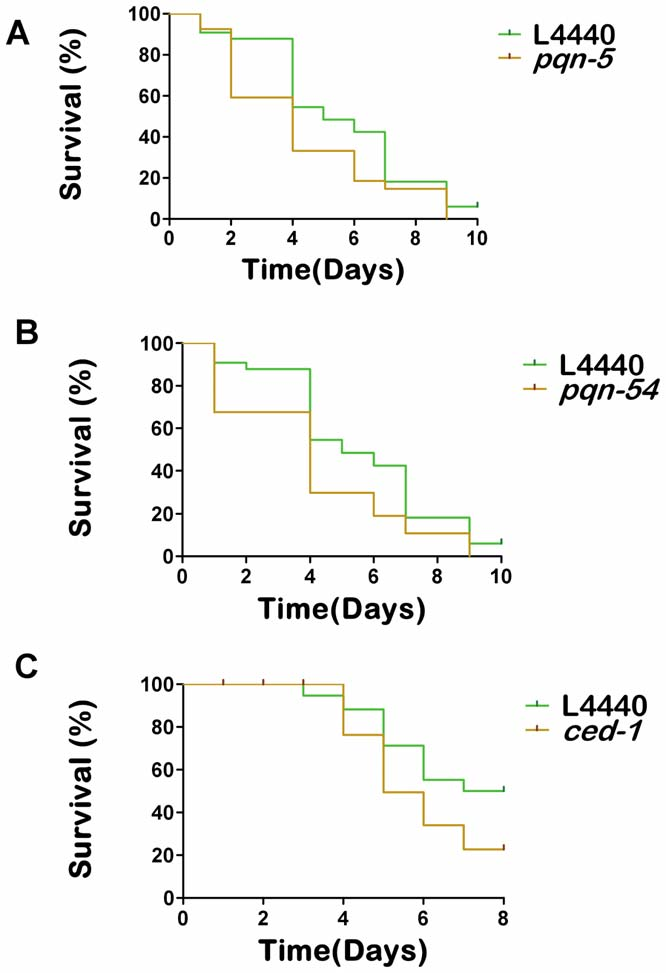
RNAi of prion-like (Q/N rich) domain protein genes *pqn-5* and *pqn-54* causes increased lethality in *C. elegans* during *V. cholerae* infection. (A) *pqn-5* RNAi survival plot, *p* = 0.04 (B) *pqn-54* RNAi survival plot, *p* = 0.02 (C) *ced-1* RNAi survival plot *p*<0.0001.

In *C. elegans* the *pqn* (prion-like glutamine [Q]/asparagines [N]) genes are identified as part of an alternative UPR pathway involved in regulating immune response against bacteria [36], [37]. A subgroup of *pqn* genes named as *abu* (activated in blocked UPR) genes, were induced to higher levels in endoplasmic reticulum (ER) stressed canonical UPR pathway mutants than in ER stressed wild type animals [38]. We found that many of *pqn*/*abu* genes are regulated through VCC in *C. elegans*. Twenty nine of seventy one *pqn* genes, and nine of eleven *abu* genes present in the *C. elegans* genome, are found to be differentially expressed in worms fed with *hlyA* deletion strains (Table 2). We knocked down seven of these genes using RNAi and found that two out of seven, namely *pqn-5* and *pqn-54*, showed increased lethality during *V. cholerae* infection in *C. elegans* (Fig. 3A, 3B). It was previously reported that PQN/ABU proteins have a distant similarity to the *C. elegans* cell corpse engulfment protein CED-1, and to a mammalian scavenger receptor of endothelial cells (hSREC), which are transmembrane cell surface proteins [39]. *ced-1* mutants are immuno-compromised, and are rapidly killed by live *Salmonella* enterica serovar Typhimurium and *E. coli*. Full-genome microarray analyses in *C. elegans* demonstrated that CED-1 upregulates expression of proteins with prion-like glutamine/asparagine (Q/N)-rich domains, which are known to be activated by ER stress and believed to aid in the unfolded protein response in *Salmonella Typhimurium* fed *C. elegans* [36]. A cluster of *ced-1* regulated genes identified by Haskins et al., found to be down-regulated in worms exposed to hemolysin deletion strains of *V. cholerae* (Table S2). We found that *ced-1* RNAi shows increased lethality when fed with *V. cholerae* wild type strain E7946 (Fig. 3C). Altogether our data suggest that PQN/ABU Unfolded Protein response genes and *ced-1* are regulated through VCC.

**Table 2:** Prion-like (Q/N rich) domain protein genes regulated by *hlyA*.

ORF NAME	GENE NAME	E7946/E7946Δ*hlyA*	CVD109/CVD110
*C03A7.4*	*pqn-5*	+3.9	+3.35
*C03A7.7*	*abu-6/pqn-6*	+3.64	+3.21
*ZC15.8*	*pqn-94*	+3.27	ND
*C03A7.14*	*abu-8*	+3.26	+2.83
*F21C10.8*	*pqn-31*	+3.2	ND
*C03A7.8*	*abu-7/pqn-7*	+3.11	+2.99
*ZK1067.7*	*pqn-95*	+1.98	+3.46
*R09B5.5*	*pqn-54*	+1.9	+2.12
*T01D1.6*	*abu-11/pqn-61*	+1.87	+1.85
*W01B11.5*	*pqn-72*	+1.81	ND
*W02A2.3*	*pqn-74*	+1.78	+1.85
*AC3.3/AC3.4*	*abu-1/pqn-2*	+1.7	+1.98
*F35A5.3*	*abu-10/pqn-33*	+1.67	+1.68
*R09F10.7*	*pqn-57*	+1.64	ND
*F39D8.1*	*pqn-36*	+1.54	ND
*D1044.3*	*pqn-25*	+1.45	ND
*T06E4.11*	*pqn-63*	+1.4	+1.73
*T23F1.6*	*pqn-71*	+1.38	ND
*Y105C5A.4*	*abu-5/pqn-77*	+1.35	ND
*Y5H2A.3*	*abu-4*	+1.34	ND
*T16G1.1*	*pqn-67*	+1.34	ND
*F31A3.1*	*abu-3*	+1.31	ND
*W03D2.1*	*pqn-75*	+1.31	ND
*C03A7.14*	*abu-8/pqn-4*	+1.28	+2.82
*M01E11.4*	*pqn-52*	−1.2	ND
*R09E10.7*	*pqn-55*	−1.23	−1.52
*F35B3.5*	*pqn-34*	−1.25	ND
*Y73B6BR.1*	*pqn-89*	−1.33	ND
*F52D1.3*	*pqn-40*	−1.79	ND
*F57B9.9*	*pqn-46*	−2.46	ND
*F29C12.1*	*pqn-32*	−3.01	ND

ND: No Difference,(+) up-regulated, (−) down-regulated.

### Determination of the regulatory genes involved in the transcriptional response against VCC

**Figure 4:**
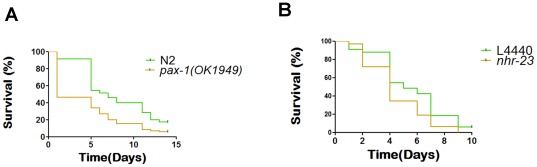
Lethality assays of the knock-downs of the FastMedusa identified immune response regulator transcription factors. (A) *pax-1(ok1949)*, *p* = 0.0013 and, (B) *nhr-23*, *p* = 0.0308 RNAi survival plots.

High throughput expression data collected from *C. elegans* in response to *V. cholerae* strains with intact and deleted *hlyA* loci provides us with a platform to search for regulatory genes involved in innate immune response, particularly the ones responsive to VCC. We used FastMEDUSA [18], a machine learning algorithm which integrates promoter sequence data, and microarray expression data, to determine the regulatory genes involve in the transcriptional response against VCC. FastMEDUSA is an open source implementation of MEDUSA [40] in C++ that uses parallel computing to decrease the execution time of MEDUSA. Using genome-wide expression changes in response to *V. cholerae* strains with intact and deleted hemolysin locus, 11 transcription factors were identified as immune regulatory genes during *V. cholerae* infection (Table 3). Five of these eleven genes are known to be expressed in *C. elegans* intestines (wormbase). We tested whether or not these transcriptional factors are required for the *C. elegans*' defense against *V. cholerae* using mutants and RNAi of these genes in lethality assay. Three genes, *pax-1*, *nhr-23* and *nhr-234*, were tested for their contribution to the organisms response to infection. We found that the *nhr-23* RNAi and the *pax-1(ok1949)* mutants exhibited increased lethality, suggesting that these genes induce the immune response in *C. elegans* (Fig. 4). *nhr-23* gene encodes a conserved nuclear hormone receptor (NHR) in *C. elegans* [41]. Human homolog of NHR-23, RAR-related orphan receptor gamma (RORγ) involves in thymopoesis [42], which is the maturation process of immune T-cells. This is the first report demonstrating a possible immune function for *nhr-23* in *C. elegans*. *nhr-234(gk865)* worms did not exhibit a significant decrease in *C. elegans* life span in lethality assay [*p* = 0.7011]. *C. elegans* has a large family of NHRs with 284 genes [43]. The *C. elegans* NHR family is a lot larger than its drosophila (18 genes), mouse (49 genes), and human (48 genes) counterparts [44]. Despite the large NHR component, *C. elegans* genome encodes only 15 conserved NHRs that belong to five of the six NHR subfamilies [41], [43], [45]. *pax-1* gene encodes a paired box transcription factor, which is involved in skeletal system development [46], and has oncogenic potential in tissue cultures and in mice [47]. This is the first report indicating a possible immune function for *pax-1*. Molecular mechanisms underlying NHR-23 and PAX-1 function in innate immune response remains to be understood.

**Table 3:** Putative regulatory transcription factors identified using the Medusa program.

Gene name	Description			GFP expression*
	CVD110	*ΔhlyA*		
*nhr-23*	✓		nuclear hormone receptor	Not known
*F22D6.2*	✓	✓	predicted Zn-finger protein	intestinal
*egl-44*		✓	similar to vertebrate TEF proteins	Intestinal
*pqm-1*	✓	✓	C2H2-type zn-finger and leucine zipper containing protein	Intestinal
*nhr-34*		✓	divergent nuclear receptor	Not known
*pax-1*	✓	✓	paired box transcription factor	Not known
*mep-1/gei-2*	✓		Zn-finger protein	Intestinal
*peb-1*	✓		DNA binding protein containing FLYWCH type Zn-finger domain	Not known
*nhr-234*	✓	✓	nuclear hormone receptor	Not known
*dhhc-2*		✓	Zn-finger protein, DHHC type	Not known
*ZK1320.3*	✓	✓	Unnamed protein	Intestine only

* GFP expression data retrieved from WormBase.

### Binding motifs identified via FastMEDUSA are associated with GO terms related to pathogen recognition, innate immune response, and inflammatory response

FastMEDUSA analysis identified DNA motifs which may constitute putative transcription factor binding sites. Top 30 motifs were identified for each; ‘CVD110 versus *V. cholerae* wild type strain E7946’ and ‘*ΔhlyA* versus E7946’ comparisons. Eighteen of these motifs were common in the two comparisons. We ran 18 common motifs against human genome using GOMO (Gene Ontology for Motifs) tool to identify the GO-terms significantly associated with the identified motifs. Using the motifs GOMO scored the promoter region of each gene in the selected organism according to its binding affinity for the motif. Using these scores and the GO annotations of the organism's genes, GOMO determined the GO terms associated with the putative target genes of the binding motif [48]. Top five GO predictions considered for each motif identified (Table 4). We found that GO terms related to pathogen recognition, innate immune response, and inflammatory response predicted as high rankers. Eleven in eighteen common motifs were found to be associated with “olfactory receptor activity” and “sensory perception of smell” functions. *C. elegans* protects itself from pathogens not only through innate immunity pathways but also through behavioral strategies such as leaving the lawn of pathogenic bacteria ([49]–[54] and our unpublished data). *C. elegans* modifies its olfactory preferences after exposure to pathogenic bacteria, avoiding odors from the pathogen, becoming more attracted to odors from familiar nonpathogenic bacteria [55]. Recently, Sun et al. showed that, in *C. elegans* ASH and ASI sensory neurons involve in the regulation of immune responses via pqn/abu UPR pathway [56]. Olfactory nervous system functions are known to be important in the murine nervous system which has the ability to detect molecules related disease or inflammation, through the vemoronasal organ [57]. Five out of eighteen common motifs were found to be associated with immune and inflammatory responses related GO terms (Table 4). One in eighteen common motifs was found to be associated with unfolded protein response, a function shown to be important in immune response against pore forming toxins [58] and to VCC (this work). Interestingly, FastMEDUSA determined *C. elegans* putative binding sites found to be enriched in the promotors of the genes belong to these categories in human genome, suggesting functional homology between *C. elegans* and human genomes.

**Table 4:** GO terms associated with binding motifs identified via FastMEDUSA.

Motif Logo	Predictions	Top 5 specific predictions
AATCGCTT	36	MF **olfactory receptor activity**
		BP **sensory perception of smell**
		MF RNA binding
		CC spliceosomal complex
		BP chromosome segregation
AATGGAC	21	MF **olfactory receptor activity**
		BP **sensory perception of smell**
		BP G-protein coupled receptor protein signaling pathway
		BP **innate immune response**
		MF **taste receptor activity**
ACAGAGG	146	CC extracellular space
		CC integral to plasma membrane
		MF calcium ion binding
		MF hormone activity
		BP cell adhesion
ACCAGAGCT	52	CC integral to plasma membrane
		CC extracellular space
		MF heme binding
		BP excretion
		CC keratin filament
ACGTGAT	57	MF **olfactory receptor activity**
		BP **sensory perception of smell**
		BP intracellular protein transport
		MF RNA binding
		BP DNA repair
ACGTTCG	408	CC nucleolus
		CC spliceosomal complex
		BP rRNA processing
		MF structural constituent of ribosome
		MF translation regulator activity
AGATTTC	28	MF **olfactory receptor activity**
		BP **sensory perception of smell**
		BP G-protein coupled receptor protein signaling pathway
		BP **innate immune response**
		BP **inflammatory response**
ATCGCTA	114	MF **olfactory receptor activity**
		BP **sensory perception of smell**
		CC mitochondrial matrix
		MF RNA binding
		BP ncRNA metabolic process
ATGCCCC	112	BP regulation of striated muscle contraction
		BP regulation of signal transduction
		CC terminal button
		BP cardiac muscle tissue morphogenesis
		CC keratin filament
ATTGTTCCA	64	MF **olfactory receptor activity**
		BP **sensory perception of smell**
		BP G-protein coupled receptor protein signaling pathway
		BP **inflammatory response**
		BP **regulation of lymphocyte mediated immunity**
CGCTAGA	158	BP nuclear mRNA splicing, via spliceosome
		CC spliceosomal complex
		CC nucleolus
		BP rRNA processing
		MF structural constituent of ribosome
CTGGAACT	0	
GAAATCT	34	MF **olfactory receptor activity**
		BP **sensory perception of smell**
		BP G-protein coupled receptor protein signaling pathway
		BP **innate immune response**
		BP **inflammatory response**
TAATCGCTG	5	MF **olfactory receptor activity**
		BP **sensory perception of smell**
TACGTTCT	37	MF **olfactory receptor activity**
		BP **sensory perception of smell**
		BP G-protein coupled receptor protein signaling pathway
		BP response to stimulus
		MF **unfolded protein binding**
TAGAACG	71	MF **olfactory receptor activity**
		BP **sensory perception of smell**
		MF RNA binding
		BP nuclear mRNA splicing, via spliceosome
		MF structural constituent of ribosome
TCAGAGAA	94	MF **olfactory receptor activity**
		BP **sensory perception of smell**
		BP G-protein coupled receptor protein signaling pathway
		CC extracellular space
		BP **immune response**
TCCAGAGG	70	CC integral to plasma membrane
		CC extracellular space
		CC proteinaceous extracellular matrix
		MF calcium ion binding
		BP regulation of monooxygenase activity

### The effect of temperature on expression of *V. cholerae* virulence factors

Many bacterial pathogens regulate the expression of virulence factors in response to changes in the environment. For example the levels of *Listeria monocytogenes* virulence gene expression depend on the amounts of the PrfA protein, which is expressed at high levels at 37°C, the temperature of the warm-blooded animal host [59]. *C. elegans* is a soil nematode with an optimal culture temperature range between 16°C and 25°C, therefore this host system is not amenable to conduct experiments at 37°C. In spite of this limitation, *C. elegans* host-pathogen interaction studies regarding human pathogens such as Salmonella species, *Staphylococcus aureus*, *E. coli* have yielded a body of *C. elegans* host response data showing high correlation to human immune response against these pathogens [60]–[64]. *V. cholerae* virulence genes are coordinately regulated by external stimuli, such as temperature, pH and osmolarity [65]. The expression of *toxR* and *toxR* regulated virulence genes including *ctxA*, was reduced between 12- and 32-fold by growth at 37°C in comparison with 30°C growth [66]. It is not known whether the decreased levels of virulence gene expression at 37°C *in vitro*, correlates with the intraintestinal expression. [66]. Effects of incubation temperature and time to the hemolytic activity of El Tor *V. cholerae* was reported by Feeley and Pittman. They found that at 35°C maximum hemolytic activity were observed at 24 hours, followed by a decline. At 30°C, maximum titres were also observed in 24 hours but the rate of decrease was less pronounced. With cultures incubated at 22°C, they measured comparable levels of maximum hemolytic activity in 48 hours, and rate of decay of activity was greatly retarded [67].

We wanted to explore the effect of temperature on the expression of *hlyA* and other virulence genes deleted in vaccine strains CVD109 and CVD110. The relative transcript abundance and expression of *V. cholerae* virulence genes *hlyA, ace*, *zot*, and *ctxA* at different temperatures was evaluated using semi-quantitative RT-PCR in *V. cholerae* wild type strain E7946. We found that all four of the genes tested were expressed at comparable levels at all the temperatures tested (Fig. S2). Our data suggest that, at our experimental conditions [22°C], *V. cholerae* virulence gene expression levels are comparable to the expression levels at the human body temperature 37°C.

In summary, we report a genome scale study regarding host responses against *V. cholerae hemolysin*. *V. cholerae hemolysin* induces expression of wide variety of immune response genes, some of which known to be responsive to other pathogenic bacteria. We found that PQN/ABU Unfolded Protein response (UPR) pathway involves in immune response against *V. cholerae hemolysin*. Using bioinformatics tools together with experimental validation, we identified transcriptional factors and transcriptional factor binding motifs involved in the immune response against VCC.

## Supporting Information

Figure S1 **qRT-PCR results for selected high ranker genes.**
(TIF)

Figure S2 **Semi-quantitative RT-PCR results showing expression levels of** ***V. cholerae*** **virulence genes at different temperatures.** Corresponding gel images are shown at the top of each column.
(TIF)

Table S1 **GO terms enriched in CVD109/CVD110 and/or E7946/Δ** ***hlyA*** **comparisons.**
(DOC)

Table S2 **Cluster of** ***C. elegans*** **CED-1 regulated genes^#^, and fold differences in expressional response to** ***hly*** **(+) versus** ***hly*** **(−)** ***V. cholerae*** **.**
(DOC)
