# Supplementary figures and images for: Genomic Analysis of Immune Response against Vibrio cholerae Hemolysin in Caenorhabditis elegans
Source: PLoS One. 2012 May 31;7(5):e38200. doi: 10.1371/journal.pone.0038200 (PMC3364981; doi:10.1371/journal.pone.0038200)

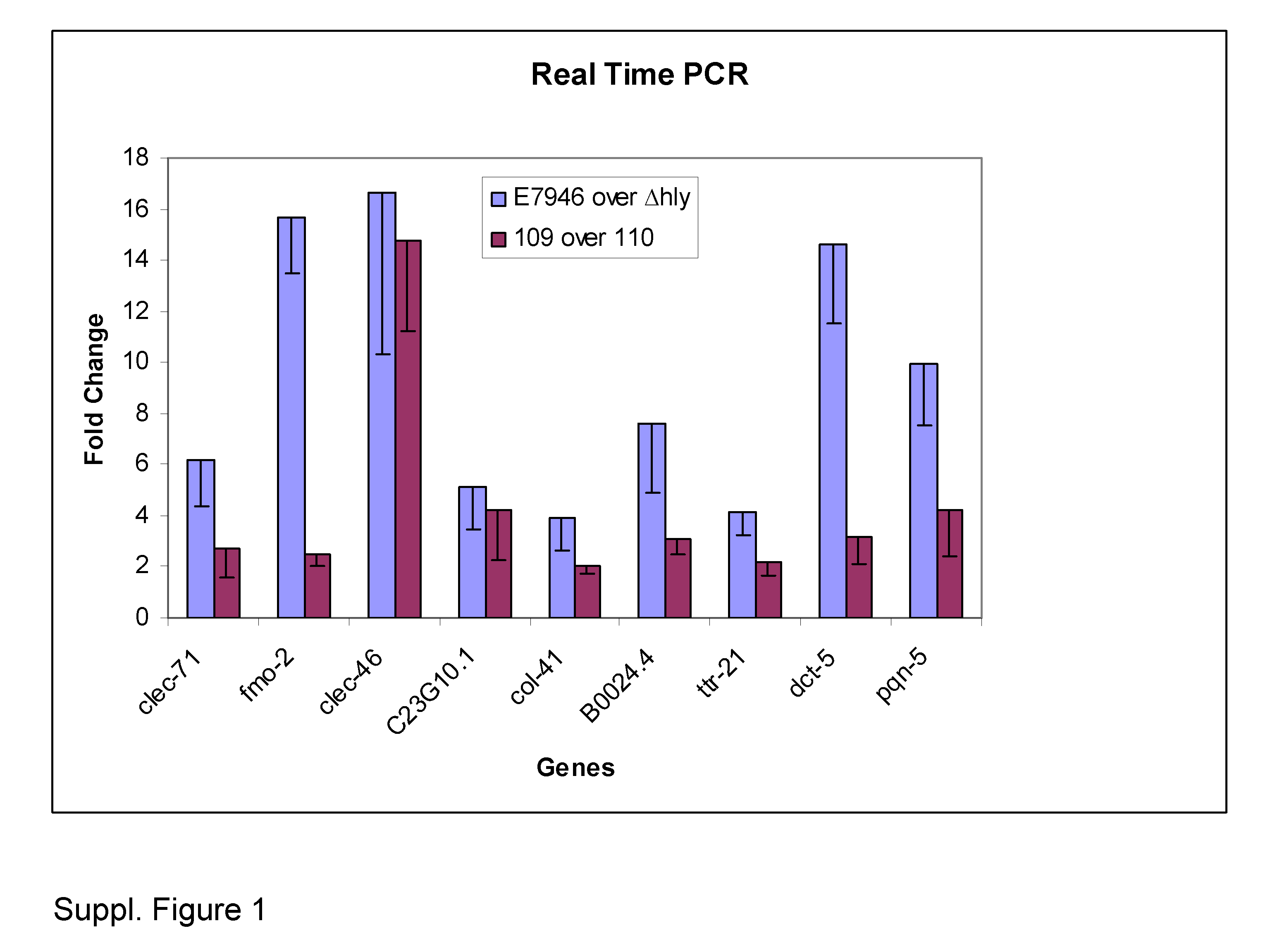

Supplement: Figure S1 — qRT-PCR results for selected high ranker genes. (TIF) [file pone.0038200.s001.tif]

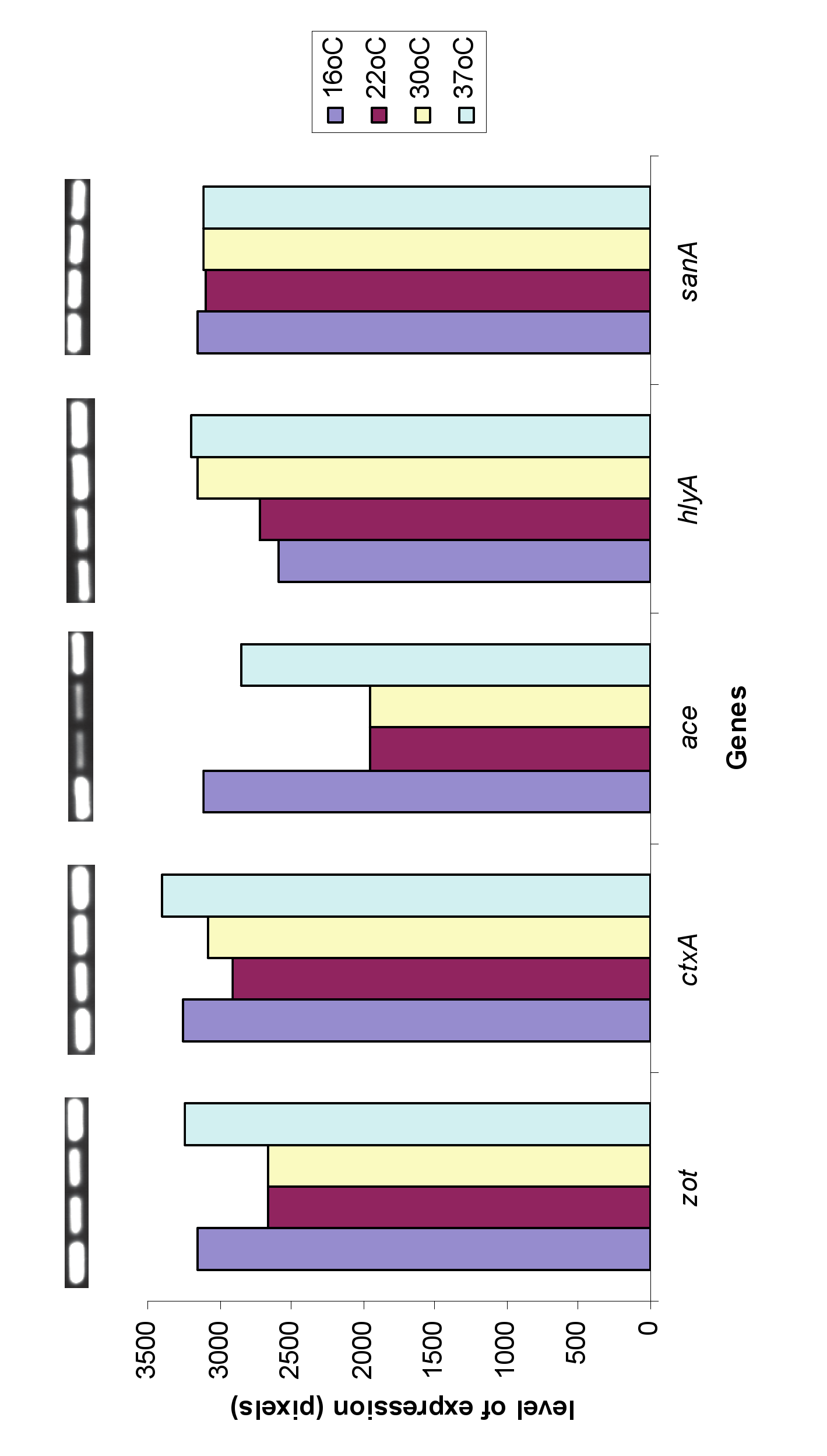

Supplement: Figure S2 — Semi-quantitative RT-PCR results showing expression levels of V. cholerae virulence genes at different temperatures. Corresponding gel images are shown at the top of each column. (TIF) [file pone.0038200.s002.tif]
