# Supplementary material for: Genomic Analysis of Immune Response against Vibrio cholerae Hemolysin in Caenorhabditis elegans
Source: PLoS One. 2012 May 31;7(5):e38200. doi: 10.1371/journal.pone.0038200 (PMC3364981; doi:10.1371/journal.pone.0038200)
Supplement: Table S1 — GO terms enriched in CVD109/CVD110 and/or E7946/Δ hlyA comparisons. (DOC) [file pone.0038200.s003.doc]

Table S1.

GO terms enriched in CVD109 / CVD110 and/or E7946 /Δ*hlyA* comparisons

| **GOTerm** | **# of genes in genome** | **# of genes in CVD109 over CVD110** | **p value** | **# of genes in E7946 over E7946** Δ***hlyA*** | **p value** |
| --- | --- | --- | --- | --- | --- |
| apoptotic cell clearance | 8 | 1 | 0.602 | 1 | 0.1816 |
| **autophagic vacuole** | 1 | 1 | **0.0529** | 1 | 0.4549 |
| autophagic vacuole assembly | 2 | 1 | 0.1378 | 1 | 0.6723 |
| autophagy | 11 | 1 | 0.759 | 3 | 0.2863 |
| **carbohydrate binding** | 16 | 5 | **1.37E-07** | 20 | **5.98E-20** |
| **carbohydrate metabolic process** | 258 | 11 | 0.2316 | 48 | **0.0003** |
| carbohydrate transport | 12 | 1 | 0.8034 | 3 | 0.3439 |
| defense response | 210 | 6 | 0.8729 | 16 | 0.2572 |
| defense response to fungus | 14 | 1 | 0.8844 | 3 | 0.4642 |
| **defense response to gram-negative bacterium** | 2 | 3 | **8.22E-11** | 4 | **4.52E-05** |
| **endoplasmic reticulum unfolded protein response** | 13 | 2 | 0.0832 | 7 | **0.0004** |
| **engulfment of apoptotic cell** | 10 | 1 | 0.7102 | 6 | **0.0007** |
| extracellular polysaccharide biosynthetic process | 10 | 1 | 0.7102 | 3 | 0.2315 |
| galactoside binding | 3 | 1 | 0.2295 | 1 | 0.838 |
| **hydrolase activity** | 227 | 10 | 0.2217 | 40 | **0.003** |
| **innate immune response** | 7 | 4 | **3.02E-09** | 6 | **5.47E-05** |
| iron ion binding | 276 | 12 | 0.1828 | 32 | 0.6625 |
| **iron ion transport** | 5 | 1 | 0.3983 | 12 | **2.89E-16** |
| lysosomal membrane | 6 | 1 | 0.4723 | 1 | 0.8314 |
| lysosome | 34 | 2 | 0.5942 | 1 | 0.2923 |
| lysozyme activity | 9 | 1 | 0.6581 | 2 | 0.64 |
| metal ion binding | 98 | 3 | 0.8783 | 10 | 0.9377 |
| metalloendopeptidase activity | 164 | 6 | 0.6803 | 22 | 0.3371 |
| metallopeptidase activity | 126 | 7 | 0.1241 | 14 | 0.9546 |
| multicellular organismal response to stress | 5 | 1 | 0.3984 | 1 | 0.9222 |
| **negative regulation of multicellular organism growth** | 440 | 2 | **0.0049** | 22 | **0.0006** |
| **nematode larval development** | 8913 | 72 | **1.58E-43** | 318 | **2.12E-130** |
| **plasma membrane** | 94 | 4 | 0.605 | 17 | **0.0535** |
| **programmed cell death** | 22 | 3 | **0.0253** | 4 | 0.4909 |
| **protein folding** | 65 | 5 | 0.0564 | 19 | **8.87E-05** |
| protein transport | 162 | 4 | 0.9925 | 17 | 0.90003 |
| proteolysis | 538 | 20 | 0.2573 | 61 | 0.6138 |
| regulation of protein catabolic process | 12 | 1 | 0.8034 | 2 | 0.8771 |
| response to drug | 13 | 1 | 0.8452 | 1 | 0.8771 |
| response to heat | 45 | 1 | 0.8224 | 7 | 0.4606 |
| hydrolase activity | 65 | 1 | 0.8221 | 5 | 0.6384 |
| **response to stress** | 16 | 3 | **0.0052** | 5 | 0.0626 |
| **secretion by cell** | 89 | 4 | 0.5367 | 17 | **0.0329** |
| sugar binding | 72 | 2 | 0.7285 | 8 | 0.969 |
| sugar:hydrogen symporter activity | 14 | 1 | 0.8844 | 3 | 0.4642 |
| **transmembrane transport** | 12 | 20 | **3.72E-91** | 91 | **1.43E-162** |
| transport | 536 | 15 | 0.8951 | 65 | 0.2985 |
| transporter activity | 266 | 8 | 0.8433 | 33 | 0.4175 |
| unfolded protein binding | 46 | 1 | 0.8429 | 10 | 0.0566 |
| vesicle-mediated transport | 60 | 4 | 0.1765 | 12 | 0.0608 |
| **zinc ion binding** | 2091 | 43 | **0.04253** | 160 | **6.22E-05** |
