# Supplementary material for: Genomic Analysis of Immune Response against Vibrio cholerae Hemolysin in Caenorhabditis elegans
Source: PLoS One. 2012 May 31;7(5):e38200. doi: 10.1371/journal.pone.0038200 (PMC3364981; doi:10.1371/journal.pone.0038200)
Supplement: Table S2 — Cluster of C. elegans CED-1 regulated genes#, and fold differences in expressional response to hly (+) versus hly (−) V. cholerae . (DOC) [file pone.0038200.s004.doc]

Table S2. Cluster of *C. elegans* CED-1 regulated genes^#^ and fold differences in expressional response to *hly*(+) versus *hly*(-) *V. cholerae*

| ORF NAME | GENE NAME | E7946/E7946Δ*hlyA* | CVD109/CVD110 |
| --- | --- | --- | --- |
| *D2096.6* | *D2096.6* | + 2.29 | + 2.71 |
| *ZK1067.7* | *pqn-95* | + 1.98 | + 3.46 |
| *F35A5.3* | *abu-10* | + 1.67 | + 1.68 |
| *AC3.3/AC3.4* | *abu-1/pqn-2* | + 1.70 | + 1.98 |
| *W02A2.3* | *pqn-74* | + 1.78 | + 1.85 |
| *C03A7.8* | *abu-7* | + 3.11 | + 2.99 |
| *C03A7.14* | *abu-8* | + 3.26 | + 2.83 |
| *C03A7.7* | *abu-6* | + 3.64 | + 3.21 |
| *F41E6.11* | *F41E6.11* | + 2.14 | + 2.16 |
| *W08E12.4* | *W08E12.4* | + 2.40 | ND |
| *Y47D3B.6* | *Y47D3B.6* | + 1.50 | + 1.59 |
| *R09B5.5* | *pqn-54* | + 1.90 | ND |
| *T01D1.6* | *abu-11* | + 1.87 | ND |
| *T05B4.3* | *phat-4* | + 1.59 | ND |
| *F20B10.3* | *F20B10.3* | + 1.99 | + 2.07 |
| *ZK662.2* | *ZK662.2* | ND | + 2.28 |
| *C03A7.4* | *pqn-5* | + 3.90 | + 3.35 |

ND : No Difference

#: Cluster of *ced-1* regulated genes list as reported in Haskins et al, 2009.
